# O057. Altered plasma adipokines concentrations in chronic migraine

**DOI:** 10.1186/1129-2377-16-S1-A56

**Published:** 2015-09-28

**Authors:** Elisa Rubino, Flora Govone, Alessandro Vacca, Annalisa Gai, Silvia Boschi, Milena Zucca, Lorenzo Pinessi, Innocenzo Rainero

**Affiliations:** Department of Neuroscience “Rita Levi Montalcini”, University of Torino, Turin, Italy

## Background

Adiponectin, leptin, and resistin are adipocyte-derived secretory factors which have functions in immune response, inflammation, endothelial function, overweight, and insulin resistance. Recently, adipokines have been suggested to contribute as inflammatory mediators of migraine. Clinical studies reported increases of adiponectin levels in migraineurs. Migraine pain severity and response to treatment in episodic migraine are associated with changes in both adiponectin and leptin concentrations. In addition, receptors for these adipokines are expressed in key structures implicated in migraine, including the hypothalamus and the cerebral microvasculature. The aim of this study was to investigate plasma concentrations of adiponectin, leptin, and resistin in patients with chronic migraine.

## Materials and methods

Twenty-seven patients with chronic migraine (20 females, 7 males; mean age 49.1±9.0 yrs) were recruited at the Department of Neuroscience “Rita Levi Montalcini”, University of Torino. All patients were diagnosed with chronic migraine according to ICHD-III beta version criteria. Thirty-four healthy subjects were used as control group (20 females, 14 males; mean age 49.2 ± 12.8 yrs). Subjects with obesity and diabetes were excluded from the study. Plasmatic levels of total adiponectin, leptin, and resistin were measured by immunoassays. Fasting glucose, insulin, total and HDL-cholesterol, triglycerides were also evaluated. The clinical characteristics of migraine were collected. Statistical analysis was performed using SPSS version 20.

## Results

Serum levels of adiponectin were significantly increased in chronic migraineurs when compared with controls (p = 0.002). After adjusting for BMI, there was no significant difference in leptin concentrations between the two groups. However, a significant positive correlation between leptin and basal insulin (r = +0.618, p = 0.002) was found in patients with chronic migraine. Finally, serum concentrations of resistin were significantly increased in female patients with chronic migraine in comparison with female controls (p = 0.03).

## Discussion

Our study provides evidence that, in addition to episodic migraine, adipokines may be involved also in chronic migraine, supporting the hypothesis that these peptides could represent potential novel biomarkers for the disease. Further studies are needed in order to better elucidate the neurobiological mechanisms underlying adipokines dysfunction in both episodic and chronic migraine.

Written informed consent to publication was obtained from the patient(s).

